# Fine Scale Analysis of Crossover and Non-Crossover and Detection of Recombination Sequence Motifs in the Honeybee (*Apis mellifera*)

**DOI:** 10.1371/journal.pone.0036229

**Published:** 2012-05-02

**Authors:** Nadia Bessoltane, Claire Toffano-Nioche, Michel Solignac, Florence Mougel

**Affiliations:** 1 Laboratoire Evolution Génomes Spéciation, CNRS, Gif-sur-Yvette, France; 2 Université Paris-Sud and CNRS, Institut de Génétique et Microbiologie, UMR8621, Orsay, France; 3 Université Paris Sud, Orsay, France; University of Minnesota, United States of America

## Abstract

**Background:**

Meiotic exchanges are non-uniformly distributed across the genome of most studied organisms. This uneven distribution suggests that recombination is initiated by specific signals and/or regulations. Some of these signals were recently identified in humans and mice. However, it is unclear whether or not sequence signals are also involved in chromosomal recombination of insects.

**Methodology:**

We analyzed recombination frequencies in the honeybee, in which genome sequencing provided a large amount of SNPs spread over the entire set of chromosomes. As the genome sequences were obtained from a pool of haploid males, which were the progeny of a single queen, an oocyte method (study of recombination on haploid males that develop from unfertilized eggs and hence are the direct reflect of female gametes haplotypes) was developed to detect recombined pairs of SNP sites. Sequences were further compared between recombinant and non-recombinant fragments to detect recombination-specific motifs.

**Conclusions:**

Recombination events between adjacent SNP sites were detected at an average distance of 92 bp and revealed the existence of high rates of recombination events. This study also shows the presence of conversion without crossover (i. e. non-crossover) events, the number of which largely outnumbers that of crossover events. Furthermore the comparison of sequences that have undergone recombination with sequences that have not, led to the discovery of sequence motifs (CGCA, GCCGC, CCGCA), which may correspond to recombination signals.

## Introduction

Crossovers are key factors for the control of co- or independent segregation of physically linked genes. They do not occur uniformly along and among chromosomes [1]–[3]. In most organisms, crossover rates (generally expressed in centimorgan per megabase – cM/Mb) vary between chromosomes: for example, in humans, from 0.96 cM/Mb for the long chromosome 1 to 2.11 cM/Mb for the small chromosome 22 [4]. The higher rate of crossovers in small chromosomes is generally explained by the necessity of at least one crossover per chromosome for proper disjunction at division I of meiosis [5], [6]. Crossover rates are also variable along the chromosomes and are generally less frequent in centromeric and telomeric regions [2], [7]–[9]. At the megabase scale, crossover variation is even more obvious and each chromosome exhibits regions with high crossover rates (called crossover “jungles”) interspersed with regions of low crossover rates (crossover “deserts”) [3], [10]. More locally, recombination rate variation can reach two orders of magnitude, which led to the definition of hot spots and cold spots of recombination [11]–[13]. Variations in crossover rate were analyzed in details in the budding yeast, mice, humans, *Drosophila pseudoobscura* and *Arabidopsis thaliana* but they are likely to be found in most organisms [4], [14]–[22].

A few studies revealed the existence of DNA motifs related to the presence of hotspots. Sequence motifs for recombination were first identified in the yeast *Schizosaccharomyces pombe* [23]. More recently, studies in humans and mice demonstrated the existence of both a sequence motif and a chromatin accessibility factor [24]–[27]. A zinc finger DNA binding protein, PRDM9, modulates hotspot usage in mice [25], [27]: It recognizes a sequence motif and changes the accessibility of chromatin by trimethylating the lysine 4 of histone H4 [24], [26]. By contrast, no specific motifs have been evidenced in *Saccharomyces cerevisiae*, in which the hypothesis of chromatin structure is uniquely invoked to explain accessibility of the DNA to the recombination machinery [28].

In the present study, we chose to explore the potential existence of sequence motifs promoting the recombination in the honeybee *Apis mellifera*. This insect model has remarkable recombination properties. First, contrary to most organisms, the 16 chromosomes exhibit similar crossover rates along their arms, despite variable lengths (ranging from 138.0 cM to 575.9 cM) and structures (15 among the 16 chromosomes are acrocentric). Second, no centromeric or telomeric effect is detected, although it could result from a lack of markers in these regions [29]. Third, crossover rates are remarkably high as compared to other organisms (e.g. 20 times higher than the human rate, [29], [30]), which makes the honeybee a powerful organism for recombination analysis. Finally, in spite of this homogeneity in crossover rates between the chromosomes, crossover rates vary slightly along the arms both at the megabase level (about twofold variation) and at the 100 kb level (up to tenfold variation). Unfortunately, the resolution of the available map (average of 93 kb between adjacent markers) was not sufficient to allow the definition of hot spots in this species.

**Figure 1:**
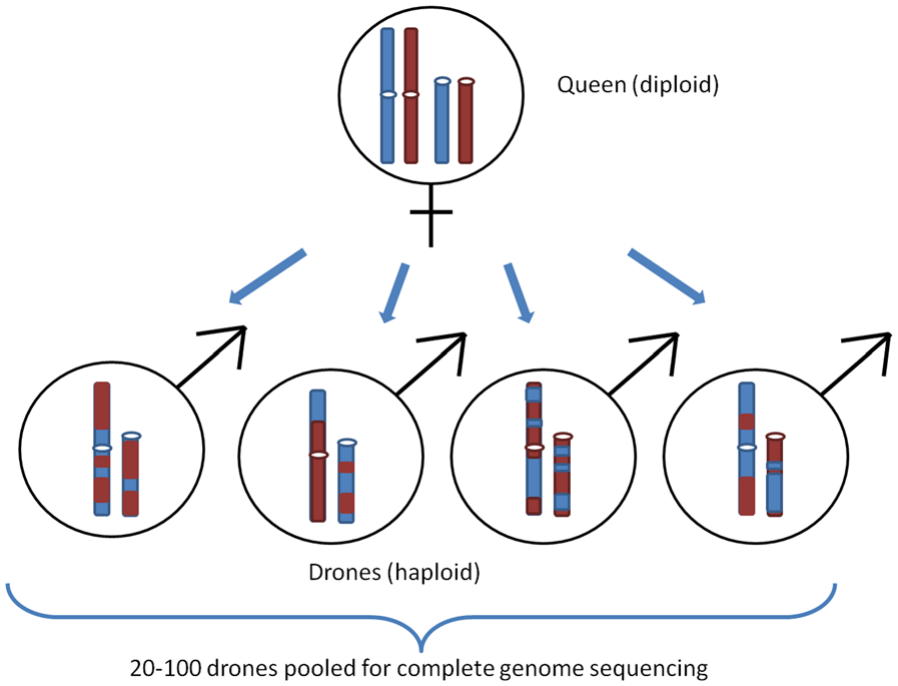
Material used for genome sequencing. The figure summarizes the strategy used for sequencing the *Apis mellifera* genome. The honeybee is a haplodiploid species in which females develop from fertilized eggs while males (drones) are issued from unfertilized eggs. DNA from twenty to one hundred drones, sons from a single queen, was pooled before Whole Genome Shotgun sequencing strategy. Genome sequence was then built from a mix of meiotic products from a single female.

To get a more precise picture of the crossover rates and localization, we took advantage of the particular approach used to sequence the honeybee genome, approach in which row sequences were obtained from many recombinant genomes [31]. The BAC library sequenced in honeybee genome sequencing project was prepared using a DNA admixture of 20–100 haploid males (drones) obtained from a single diploid queen (Hugh Robertson, pers. comm.). In the honey bee, the drones developed from unfertilized eggs and thus, each drone represents the amplified DNA of a single female gamete (Figure 1). Therefore, using a panel of brother drones would correspond to an “oocyte method” (study of recombination on female gametes) similar to the “sperm method” (study of recombination directly on male gametes or spermatozoids) used in humans [32], [33]. The set of brother drones bears different haplotypes. Most of them correspond to the maternal haplotypes while few of them correspond to recombinant ones. To detect these recombinant haplotypes, we used as markers the SNP defined from the complete sequencing project. A consequence of the DNA source used for the bee genome sequencing is that these SNPs reflect the heterozygozity of the queen. Analysis of the panel of reads (raw sequencing products) covering the same region of the genome allows reconstructing the two parental haplotypes present in the queen as well as identifying the recombinant haplotype(s) of the progeny (if any). Recombination events were identified with a resolution defined by the average distance between two adjacent SNPs that is 92 bp. Fine study of these recombinant fragments suggests that non-crossover (that is conversion without crossover) is very frequent in honeybee genome. In a second step, we analyzed the sequences delineated by two recombinant SNPs to search for specific motifs that could promote recombination. Oligonucleotide composition of these recombinant fragments was compared to the composition of non-recombinant fragments selected in a similar fashion. Candidate motifs were further quantified in DNA fragments for which crossover rates is known. Positive correlation was found with three sequence motifs (CGCA, GCCGC, CCGCA), which may correspond to recombination signals.

## Results

### Localization of recombination events

Localization of recombination events was investigated between SNPs located on mapped scaffolds. We compared the number of recombination events actually observed with the number of crossover events which would be expected based on the length of the genetic map [29]. Over the sixteen chromosomes of the honeybee, 362,456 SNP pairs could be studied as they were covered by at least three reads (Table 1). The physical size of this dataset corresponds to 24% of the assembled complete sequence. The rest (76%) of the nucleotides in the assembled genome was eliminated for various reasons: a lack of heterozygozity (i.e. lack of SNP); a large distance between 2 consecutive SNPs implying that too few reads covered both SNPs; a poor coverage by reads in some regions of the genome (See Material and Methods, section 2 and 3).

**Table 1:** Summary of recombinant and non recombinant set.

		Testable SNP pairs		Recombinant pairs		Non recombinant pairs	
Linkage group	Length (bp)	Number	Length (bp)	Number	Length (bp)	Number	Length (bp)
1	22,954,376	52,488	5,749,440	65	5,619	12,410	847,421
2	12,965,785	24,583	2,921,124	19	1,358	5,550	387,581
3	10,891,916	26,549	2,821,662	34	2,658	6,755	462,276
4	9,896,202	21,230	2,397,447	25	1,614	4,571	324,098
5	12,136,189	24,305	2,531,713	23	1,959	6,280	412,814
6	12,781,788	27,094	2,847,285	30	2,658	6,953	468,254
7	8,474,240	18,898	1,891,977	21	2,370	4,853	311,174
8	9,702,794	21,638	2,145,767	31	3,139	5,512	343,769
9	9,282,195	22,437	2,384,528	41	3,762	5,523	369,087
10	9,590,700	19,643	2,240,080	13	1,032	4,744	323,707
11	11,126,330	26,192	2,728,042	47	5,215	6,281	417,273
12	8,382,753	20,060	2,307,638	31	2,734	4,212	282,116
13	8,179,068	18,345	2,043,979	20	1,876	4,125	274,731
14	7,468,479	16,137	1,850,922	12	1,086	3,522	237,501
15	6,756,270	12,215	1,306,254	17	2,001	2,844	189,096
16	5,181,066	10,642	1,086,928	15	2,009	2,721	160,420
All	165,770,151	362,456	39,254,786	444	41,090	86,856	5,811,318

Total length of each linkage group and of the whole genome is given (2nd column). “Testable SNP pairs” correspond to SNP pairs for which enough reads overlap to check for recombination. “Number” gives the number of SNP pair and “Length” sums the cumulative size of all the pairs over one linkage group or over the whole genome. “Recombinant pairs” and “Non recombinant pairs” correspond to the final set validated by 6 SNP (see text and figure 1).

**Figure 2:**
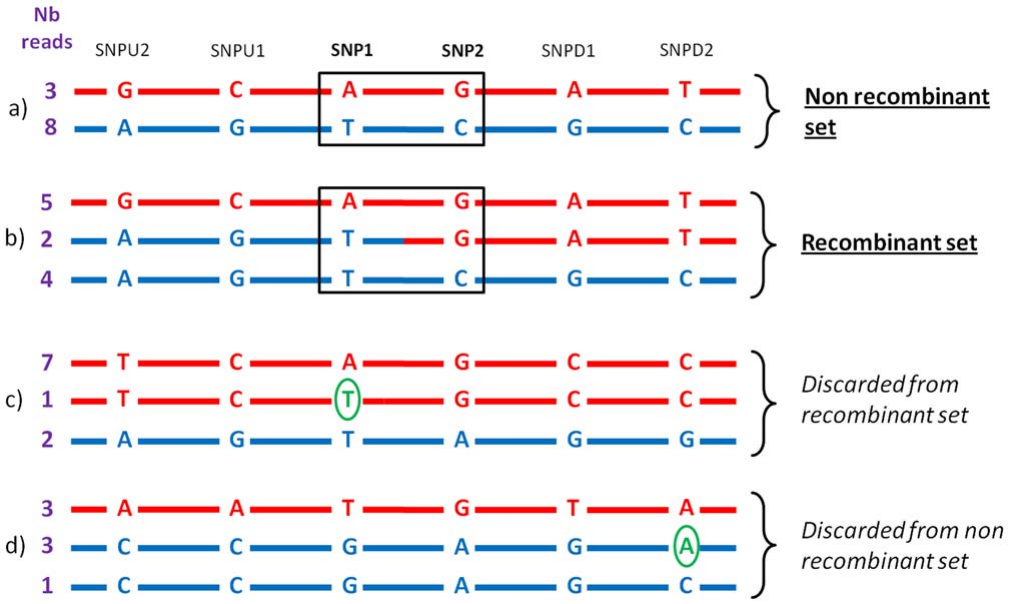
Principle of recombination detection. The number of haplotypes was computed for each pair of successive SNPs (here called SNP1 and SNP2). When 2 haplotypes were observed, they are supposed to correspond to maternal ones (a and d). When 3 or 4 haplotypes were observed, a recombination event was suspected between the two SNPs (b and c, blue and red colors indicate the maternal phases). The pair was conserved in the recombinant set if the haplotypes were confirmed by 4 supplementary flanking SNPs, two upstream SNPU1, SNPU2, and two downstream SNPD1 and SNPD2 (b) or was discarded otherwise (c). The same strategy was applied to collect non-recombinant SNP pairs showing 2 haplotypes (a and d). “Nb reads” indicates the number of observed reads in each haplotype.

Based on the numbers of haplotypes observed for each given pair of SNPs, the 362,456 SNP pairs could be divided into two groups: 61,929 SNP pairs showing possible recombination (3 or 4 haplotypes observed) and 300,527 SNP pairs showing no evidence of recombination (2 haplotypes only). However, this first screen clearly overestimates the number of recombinant pairs for at least two reasons: (i) sequencing errors in individual read sequences, which are corrected when contigs are assembled to build the consensus genome sequence; (ii) point mutations in the DNA of the cloned sequence of one single drone will appear as a “recombinant” pair (point mutations in the germ line of the mother queen also). To remove such sequencing errors or drone-specific mutations, we validated the observed haplotypes by observation of four flanking SNPs: Two located upstream of the first SNP (SNPU1 and SNPU2, Figure 2) and two located downstream of the second one (SNPD1 and SNPD2, Figure 2). We checked that for each haplotype defined by the two SNPs SNP1 and SNP2, only one haplotype was observed from the surrounding SNP and that only 2 haplotypes (maternal haplotypes) were observed on each side of the pair (Figure 2). An example is given in Figure 2b where 3 haplotypes “AG”, “TG” and “TC” are observed for SNP1 and SNP2. For each of them, only one haplotype is observed for SNPU1, SNPU2, SNPD1 and SNPD2 over the 11 overlapping reads. Furthermore, only two haplotypes are observed over SNPU1, SNPU2 and SNP1: “GCA” and “AGT”. The same is true on the other side of the recombination event where haplotypes “GAT” and “CGC” are the only ones detected over SNP2, SNPD1 and SNPD2. In this case, the recombination event is confirmed. This validation step resulted in the elimination of many SNP pairs (Fig. 2c and 2d). Out of the original set of 362,456 SNP pairs, only 87,300 pairs were selected. In this final set, 444 pairs were most likely true recombinants (with a distance between successive SNP ranging from 1 to 543 nucleotides, nt, average: 92.5±96 nt) and 86,856 pairs were non-recombinants (with a distance between successive SNP ranging from 1 to 858 nt, average: 66.9±87 nt). In total, the reduction was far more dramatic for recombinant pairs as only 0.7% of the initial set of 61,929 SNP pairs was kept comparing to 29% of the non-recombinant set of 300,527 SNP pairs. Elimination due to read coverage is supposed to influence recombinant and non-recombinant pairs in the same way. However, elimination due to sequencing error is more likely to be found in SNP pairs showing 3 or 4 haplotypes in the first screen because these supplementary haplotypes will precisely reveal sequencing errors. The final retained set comprised 5,852,408 bp corresponding to 3.5% of the nucleotides in the analyzed genome assembly.

### Honeybees exhibit high levels of meiotic exchanges

The genetic length of the honeybee genome was estimated to be 4,114.5 cM [29], and thus, there are on average 41.1 crossover events at each meiosis. The genome sequence has been built from multiple drones (20–100, Hugh Robertson, pers. comm.) derived from a single queen. The effective size of the progeny genotyped at each SNP site can be estimated from the coverage observed on the SNP retained and from the effective number of different drones sequenced given this coverage. The selection process lead to an increase of the coverage in the final set which reached 12.3× when the mean over the whole genome was 7.5× [31]. Among these 12.3 sequences, multiple copies of a single drone fragment can be observed by chance. We estimated the effective number of different drones sequenced in each point for the two extreme values of number of drones used in sequencing project: 20 and 100 (See Material and Methods for further details). We obtained an estimate of 9.2 and 11.4 different drones sequenced. In other words, at each position, between 9.2 and 11.4 sequences issued from different drones are available. If all the genome could be scanned, we could expect to detect between 41.1 * 9.2 = 378 and 41.1 * 11.4 = 467 crossover events. As this study screened only 3.5% of the genome we expect to detect only between 13.2 and 16.3 crossover events.

Among the final set of analyzed sequences, we found 444 SNP pairs surrounding recombination events. Among them, 442 showed 3 haplotypes and 2 showed 4 haplotypes. When 4 haplotypes are observed, we can conclude that two independent recombination events occurred in the same interval defined by the SNP pair. When only 3 haplotypes are observed, we cannot be sure whether they derived from a single or from more recombination events. However, it is likely that there were as many recombinations in two different drones sharing the same haplotype as in two different haplotypes. We can thus estimate that we identified about 440 pairs suffering one recombination event and 4 pairs suffering two recombination events leading to an estimate of 448 recombination events in our sample.

### Non-crossover events are more frequent than crossover events

**Figure 3:**
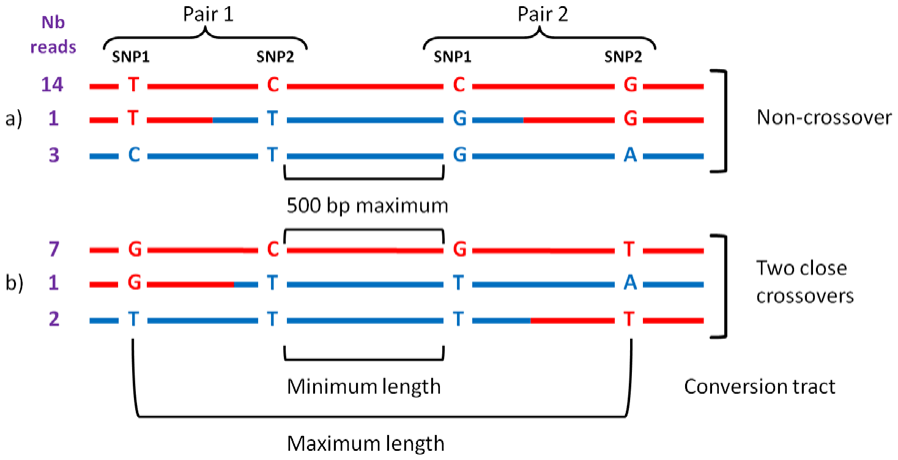
Distinguishing non-crossover from close crossover.

The above number of recombination events is between 27 (448/16.2) and 34 (448/13.2) i. e. about 30 times higher than the number of crossover events expected from the length of the genetic map. The genetic length was obtained from markers spaced every 93 kb on average so that genetic mapping could not detect conversion events not associated with crossover (non-crossover events, NCO). Among the above 444 recombinant SNP pairs, 22 were located at less than 500 bp from another pair, a distance covered by single reads which have a mean size of 600 bp. We could thus study 11 clusters of two recombinant SNP pairs to check whether the same read was involved in the two recombination events or whether different reads were concerned. The principle for solving this issue is shown in figure 3. Over the 11 clusters of recombinant SNP pairs, two resulted from two independent crossovers and 8 resulted from non-crossover (Table 2). The last cluster was at the upper limit of the sequence size (671 bp between first SNP of pair 1 and second SNP of pair 2) leading to a lack of reads covering the 4 SNP. This fine scale analysis allowed deciphering the presence of non-crossover in honeybees. It also showed that in bees non-crossovers are far more frequent than crossovers. Finally, the high number of non-crossovers likely explains the discrepancy between the recombination events detected in our genome scan and the crossover events obtained by genetic mapping [29].

**Table 2:** Fine study of close recombination events.

	Pair1		Pair2					
Scaffold	SNP1	SNP2	SNP1	SNP2	Span (bp)	Nb covering read	Event	Conversion tract (bp)
Group2.7	AMB-00232006	AMB-00232005	AMB-00232003	AMB-00231998	125	18	non-crossover	32–125
Group2.19	AMB-00067193	AMB-00067192	AMB-00067188	AMB-00067186	346	4	non-crossover	182–346
Group6.33	AMB-00652988	AMB-00652989	AMB-00652994	AMB-00652995	195	12	non-crossover	123–195
Group8.15	AMB-00436854	AMB-00436857	AMB-00436865	AMB-00436869	671	0	-	
Group8.21	AMB-00942680	AMB-00942681	AMB-00942683	AMB-00942685	158	10	2 independent crossovers	
Group9.4	AMB-00002147	AMB-00002148	AMB-00002160	AMB-00002162	259	15	non-crossover	230–259
Group9.12	AMB-00260794	AMB-00260795	AMB-00260798	AMB-00260799	106	8	non-crossover	80–106
Group9.14	AMB-01117814	AMB-01117815	AMB-01117817	AMB-01117820	185	7	2 independent crossovers	
Group9.16	AMB-00011356	AMB-00011357	AMB-00011362	AMB-00011363	322	6	non-crossover	144–322
Group11.23	AMB-00345683	AMB-00345684	AMB-00345687	AMB-00345688	187	6	non-crossover	82–187
Group12.8	AMB-00717181	AMB-00717185	AMB-00717200	AMB-00717204	665	4	non-crossover	341–665

The 11 couples of SNP pairs with less than 500 bp between the two pairs are shown. The identifiers of the 4 SNPs concerned are given as well as the scaffold they come from. The “span” column corresponds to the distance between the first SNP of the first pair to the second SNP of the second pair. “Nb covering read” indicates the number of reads covering the 4 SNPs studied. “Event” is the conclusion of a fine examination of haplotypes (see the text for further details).

### Recombination signal motifs in honeybee

We next used the recombinant set to search for specific motifs. Sequences between two recombinant SNPs were extracted and compared to sequences between two non-recombinant ones. The recombinant fragments were used to produce two datasets. They were either extracted as is (*intervening* dataset with 41 fragments shorter than 10 nt discarded) or extended by 40 nt on each side (*extended* dataset, see Material and Method section 4). The extension was applied independently of the fragment size because we do not know if the recombination occurred in the middle of the fragment or close to one of the surrounding SNP. Through this extension process, the smallest fragments (encompassing 1 to 10 nt between SNP) were enlarged to 81 to 90 nt and became suitable for a study of oligonucleotide occurrence. Recombinant sequences as well as non recombinant ones used for comparison were masked for repeat and low complexity sequences and search was performed preventing overlapping matches (noov option in the RSAT suite, see Material and Methods). Table 3 summarizes the motifs found for both datasets. One motif is mentioned despite a negative occ-sig (GCCGC) with the options applied. We chose to keep it in mind for several reasons: i) it was significant without applying noov option, ii) it has interesting overlapping properties with other significant motifs (CGCA and CCGCA), and iii) it is an auto overlapping motif and could be excluded only for technical reasons. All detected motifs were overrepresented in the recombinant set. Occurrence of the motifs within the 444 fragments is indicated in the last column of table 3.

**Table 3:** Motifs overrepresented in the recombinant set.

Word size	Motif	Occ-sig		Number of sequences	Correlation with crossover rate	
		intervening	extended		Corr	Sign
L4	aaaa|tttt	0.54	-	420	−0.402	***
	cgca|tgcg	0.35	-	174	***0.357***	***
L5	aaaac|gtttt		1.23	232	−0.252	***
	aaaaa|ttttt		0.5	343	−0.376	***
	taaaa|tttta		0.92	333	−0.406	***
	tgaaa|tttca		0.27	306	−0.385	***
	ataaa|tttat		0.26	324	−0.381	***
	aaaga|tcttt		0.01	288	−0.105	**
	gccgc|gcggc	−0.09		47	***0.433***	***
	ccgca|tgcgg	0.5		44	***0.309***	***
L7	gaacaga|tctgttc		0.07	21	−0.021	NS
L8	actgttcc|ggaacagt		0.07	9	***0.062***	NS

Motifs indicated were retained if they were 4 nt or more for intervening dataset and 5 nt or more for extended dataset. Occ-sig statistics given by RSAT are indicated only if they are positive except for GCCGC (see text). “Number of sequence” gives the number of recombinant pairs in which the motif could be found at least one time over the 444 recombinant fragments. “Corr” gives Pearson correlation coefficient between crossover rate inferred from the published genetic map and motif occurence (positive values are in bold and italics). “Sign” indicates whether the coefficient is statistically different from 0: “NS” non significant, “***” P-value<0.001, “**” P-value<0.01.

Despite positive occ-sig for most of the motifs shown, only weak signals are detected. To further test whether they could have some influence on crossover, we decided to compare their relative occurrence in DNA fragments with the crossover rates measured from published genetic map. Pearson correlation coefficients are also given in table 3 as well as the significance of the coefficients after correction for multiple testing. Except for the largest motifs, all the correlations are significant. However, this correlation is negative for A/T rich motifs indicating that they are less frequent in fragments with high crossover rates than in fragments with low crossover rates. On the opposite, correlation is positive for the three G/C rich motifs which indicate they are good candidates as crossover signals.

## Discussion

### First evidence of non-crossover conversion in honeybees

Our genomic analysis reveals for the first time the occurrence of NCO in honeybees. This process has been described for a long time in fungi as an alternative process to crossover [34]. Estimation of the relative frequency of CO and NCO in a genome has however been approached more recently in a wide variety of species. The ratio NCO/CO could be estimated at 0.3 directly from meiosis products in *Saccharomyces cerevisiae* [19], [35]. In other model species with larger genomes it was deduced from linkage disequilibrium analysis [36], [37] or from the comparison of the number of double strand breaks initiating recombination and the number of CO [38], [39]. The NCO/CO ratio reaches values between 5 and 20 in plants and metazoans. Some estimations in humans based on linkage disequilibrium even rose to 25–125 when the mean length of the conversion tract was supposed to be short (100 bp). The ratio of 30 (total number of recombination events detected/expected number of crossovers) observed in the present study is in total agreement with the estimations obtained in animals and plants.

As suggested above, the influence of NCO events on linkage disequilibrium depends both on their frequency and length. In *Saccharomyces cerevisiae*, the length of the conversion tracts was estimated at 1.8 kb over 2000 events observed in a wide genomic study [19]. Estimations in other model organisms rely on the fine study of a few recombination hot spots and are therefore only partial. They indicate however that conversion tracts are probably smaller in animals than in yeasts. The observed length ranged from 50 to 300 bp in human [40], 10 bp to 290 bp in mice [20] and 350 bp to 700 bp in fly [41]. In honeybees, the observed values between 32 bp to 671 bp (average: 214 bp) fit better with the values observed in metazoans than in yeast. However, because the conversion has to be included in reads, the estimation could be performed only for short conversion tracts. Therefore there could be some bias in our estimation of this length. The real value could be larger than the observed one.

NCO events are known to influence allelic diversity and to decrease linkage disequilibrium locally [42], [43]. Their influence on genetic shuffling thus strengthens the role of crossover. Interestingly, crossover rate of honeybee is now well established as one of the highest among metazoan [29], [44], [45]. The possible causes of this high rate have been widely discussed but the reasoning could be extended to NCO rate. Hypothesis mainly retained to explain the high crossover rate are linked to the eusocial status of the species [46]–[48]. The genotypic diversity is increased in the progeny by the shuffling realized by crossovers: It is supposed to favor disease resistance at the colony level and the division of labor between individuals [46]. Furthermore, crossover are supposed to increase the homogeneity of the relatedness between workers of the same colony favoring the social organization under kin selection hypothesis [47]. Finally the high crossover rate is proposed to counterbalance the small effective population size of honeybees (e.g. size of the population of sexual individuals that effectively reproduce) which would otherwise hamper the selection process [30]. Because the impact of NCO is mainly local, it would be interesting to test its influence on genotypic diversity, relatedness, or response to selection. Furthermore, it would also be very informative to test whether NCO rate is also high in other eusocial species.

### Sequence motifs for recombination

Our study allowed proposing twelve motifs associated with recombination. These motifs can be divided in two well distinct groups: A/T rich motifs found with the extended dataset and G/C rich motifs found with the intervening one. The only exception is AAAA which is retained only for intervening set. However, this motif showed also a positive occ-sig (3.93) in the extended set but is not mentioned due to the high level of false positive oligonucleotides of length 4 nt with this dataset (see strategy described in Material and Methods section).

The A/T rich motifs are very frequent in the recombinant set: AAAAC and AAAAA were observed in 52% and 77% of the recombinant fragments respectively. Despite their high frequency within recombinant fragments, they are probably poorly specific in an A/T rich genome as that of the honeybee [31]. A similar study in *Drosophila pseudoobscura*, proposed various motifs from the comparison of sequences with high recombination rates and sequences with low recombination rates [14]. Most of them were A/T rich motifs such as TTAAAA or AAATG. A further correlation analysis of motifs with recombination rate eliminated most of these candidates. Correlation analysis presented in table 3 indicates that all these A/T rich motifs are negatively correlated with crossover rate. This result is not sufficient however to eliminate the A/T rich motifs as recombination signals. They could be specific to non-crossover events.

The group of G/C rich motifs comprises three motifs compatibles to form larger motifs: CGCA is included in CCGCA; the motifs GCCGC and CCGCA can be associated to obtain the 6 bp motif GCCGCA. In fact, among the 44 fragments presenting the CCGCA motif and the 47 ones presenting the GCCGC motif, 20 fragments show both motifs, and 13 of them contain the 6 bp motif. However, the size of the intervening dataset is yet too small to identify significant motifs larger than 5 bp. The whole recombinant sample comprises 41,090 bp, while there are 4,096 (4^6^) possible hexanucleotides or 16384 (4^7^) possible heptanucleotides. Consequently, motifs larger than 5 bp were not detected with enough statistical power. The compatibility of the G/C rich motifs nevertheless strengthens their validity.

On the other hand, G/C rich motifs are observed in low frequency among the recombinant fragments compared to A/T rich motifs. The difference could have been due to specificity for CO sites versus NCO sites. The positive correlation observed between G/C rich motifs and crossover rate compared to the negative correlation with A/T rich motifs argues for this hypothesis. However, the specificity is probably not so strict as numerous fragments contained both A/T rich and G/C rich motifs.

The low frequency of G/C rich motifs however does not exclude them as potential recombination signals. When we cumulate fragments displaying only CCGCA, only GCCGC or both CCGCA and GCCGC, we obtain 71 fragments, that is, 16% of the recombinants. It should be noted that the first motif described in humans, CCTCCCT, was only observed in 11% of the 25,000 recombination hotspots studied [49], which is similar to what we describe here. The authors increased the representativeness of their motif when they included some degeneracy to lengthen it: CCNCCNTNNCCNC is observed in 40% of the human hotspots [50]. The observation of the two motifs CCGCA and GCCGC alone in several recombinant fragments suggests that degeneracy may also occur in honeybee motifs. Unfortunately, this hypothesis cannot be tested with the small dataset available in this study.

The low frequency of the G/C rich motifs could explain why the motifs are not found in the *extended* set: the signal could be diluted with extension, which induces doubling of the sequence space. Interestingly, when the MEME [51] tool for motif detection was used, only the GCCGC motif was found and it was detected only in the *intervening* dataset. The detection of this motif with two different analysis tools strengthens its potential validity.

Finally, the low frequency of detected motifs could also derive from the existence of different categories of motifs in different recombination sites. For example in the fission yeast, *Schizosaccharomyces pombe*, four different motif categories with different levels of degeneracy were shown to influence recombination [52]. The motifs described here could represent only one of the categories of recombination signals existing in honeybees.

## Materials and Methods

### Sequencing Data

The honeybee genome sequences were downloaded from the FTP site of the Human Genome Sequencing Center, which was in charge of the honeybee sequencing project (HGSC, ftp.hgsc.bcm.tmc.edu/pub/data/Amellifera/). One million honeybees SNP identified from the sequencing project were also downloaded from the HGSC ftp site (ftp.hgsc.bcm.tmc.edu/pub/data/Amellifera/snp/amel_v3_asm_snps.dbsnps.gz). We worked with version 3.0 of the genome used to define these SNP. Number of reads and thus coverage is the same as in the published 4.0 version of the sequence [31]. The difference between the two genome versions mainly resides in the mapped portions of the sequence. Sequence of the scaffolds, coordinates and orientation of the contigs on the scaffold (ftp.hgsc.bcm.tmc.edu/pub/data/Amellifera/fasta/Amel20050501-freeze/Scaffold_contigs_20050501.agp) and whole read sample (ftp.hgsc.bcm.tmc.edu/pub/data/Amellifera/fasta/Amel20030815-reads/) were also downloaded from the HGSC ftp site.

### Associating reads and SNPs: definition of allelic variants

SNP mainly reflect the heterozygosity of the queen but they also result from sequencing errors or pre- and post-meiotic mutations. We therefore eliminated all SNP showing more than two variants. Furthermore, we selected SNPs for which both variants were each observed at least twice with high quality standard defined by HSGC: they applied the NQS criterion (Neighbourhood Quality Standard defined from [53]) 5 bases upstream and 5 bases downstream of each mismatch. Furthermore, only mismatches from high quality bases (quality score > = 20) were included by HSGC in the SNP set downloaded for the present study. All the reads available at HGSC (3.8 millions) were located on the v3.0 sequence assembly using the Megablast software with default parameters [54]. Alignments covering at least 70% of the read sequence with a minimum of 80% identity were conserved. These thresholds were respectively chosen because of the poor quality of sequence extremities (for which poor quality alignment is not disabling) and to conserve sequence divergence due to the queen heterozygosity. Comparison of the coordinates of reads with those of SNP on the sequence assembly allowed defining a set of covering reads for each SNP. For each set, alleles carried by the reads were determined through a multiple alignment approach. The read sequences centered on the SNP were aligned with a sequence of 200 nt around the SNP position extracted from the scaffold (100 nt upstream and 100 nt downstream) using DIALIGN2.2 [55]. As overlapping between reads was sometimes small, only the part of read sequence matching the 200 nt around the SNP was conserved to avoid misalignments. The alleles deduced from the multiple alignments were thus confronted with those described by HGSC and the reads were conserved only when alleles were corresponding. This approach allowed identifying for each SNP the covering reads and the allele they bear.

### Localization of recombination events

The principle of the search for recombination events is summarized in figure 2. Scaffolds were scanned for each pair of successive SNP. Allelic associations (or haplotypes) at both SNP were computed. When only two haplotypes were observed, we assumed that they corresponded to maternal haplotypes and that no recombination occurred between the two SNPs. The existence of three or four haplotypes was considered as corresponding to at least one recombination event. To eliminate false positives, both cases were subsequently confronted to information obtained from four additional SNPs: two SNPs upstream of the first SNP and two SNPs downstream of the second one. To add a SNP pair to the recombinant set, each of the 3 or 4 haplotypes had to be confirmed by at least one read covering 3 SNPs upstream the potential recombination event (SNPU2, SNPU1, and SNP1) and at least one read covering the 3 SNPs downstream the potential recombination event (SNPD2, SNPD1, and SNP2). Similarly, when the two maternal haplotypes were confirmed by the surrounding SNPs, the pair was added to the non-recombinant set. Many candidate pairs for recombinant and non-recombinant set were therefore discarded because of the lack of SNP at small distance or from the lack of read coverage of the surrounding SNPs preventing the validation of each haplotype. Some other ones were highly covered but revealed some sequencing errors in one of the reads and were also eliminated (figure 2c and 2d). This approach led to the selection of 444 SNP pairs in the recombinant set and of 86,856 SNP pairs in the non-recombinant set.

### Estimate of the expected number of crossovers

We intended to compare the number of recombination events detected with the number of crossovers expected from the genetic length of the honeybee genome (41.1 Morgan, M) and the progeny size studied. The number of males used for genome sequencing was comprised between 20 and 100. The expected crossover number was calculated from both extreme values. In each case we assumed that each drone contributed in equal proportion to the DNA pool used for sequencing strategy and that the DNA copy number from each drone was very large. Under these two hypotheses we can model the sequencing at each specific position as a sampling with replacement of K individual sequences within a set where the probability to draw a sequence from a specific drone is 1/N, K being the mean coverage of the sequence and N being the number of males used to make the DNA pool (20 or 100). Then the mathematical expectation of the number of raw sequences coming from different drones in a sample of size K is N-N(1-1/N)^K^. The mean coverage was estimated at 12.3 over the selected set of SNP. Taking the round value of 12 and 20 to 100 drones to form the DNA pool, we obtained an estimate of progeny size between 9.2 and 11.4 (*e.g.* the number of sequence issued from different drones). Consequently, over the whole genome the expected number of crossover is estimated between 378 and 467.

### Detection of motifs involved in recombination

Sequences corresponding to the recombinant set were extracted from scaffold sequences in two ways: i) *intervening* dataset: the strict DNA fragment between the two SNP was extracted but 41 pairs which were shorter than 10 nt were removed, ii) *extended* dataset: the DNA fragment between the two SNP was extracted with an extension of 40 nt on each side of the fragment. In this last case, the 444 pairs were extracted.

In contrast to the recombinant set, numerous pairs in the non-recombinant set were contiguous. In this case these sequences were concatenated in single fragments. The resulting non-recombinant set comprised 19,338 sequence fragments. Hereafter it is referred as the “reference set”. It was used as a basis for motif frequency in comparison with recombinant set. The non-recombinant set was also used to build random negative controls. Negative controls were similar in numbers of sequences and in sequence size to the recombinant set. One thousand such negative controls were sampled with similar fragment size than the intervening set as well as 1000 negative controls similar to the extended set. These two set were used to check whether observed data fit the theoretical model [56].

For motif detection *per se*, we used the software suite RSAT (Regulatory Sequence Analysis Tool) and more specifically *Oligo-analysis* tool to compare the recombinant set with the reference one [57], [58]. This tool searches for over- or under-represented oligonucleotides in the test set (the recombinant set in our case) with comparison to a reference set. The distribution of the expected occurrences of the oligonucleotides is supposed to follow a binomial distribution. *Oligo-analysis* calculates for each motif a P-value which corresponds to the probability to get the observed or highest occurrence of the motif under consideration if the frequency is the same than in the reference set. This P-value is multiplied by the number of different words of the same length to correct for multiple testing, leading to the E-value. An E-value of 1 indicates that we could expect 1 occurrence at random in the dataset analyzed. The statistic given by *Oligo-analysis* (occ-sig) is derived from this E-value: occ-sig = Log10(E-value). Positive values of occ-sig should correspond to less than 1 expected false positive. Beside the *Oligo-analysis* tool used, other refinements were tested to check the best fit to the theoretical model. RSAT allows detecting and eliminating duplicated sequences (purge option). It can also allow or prevent overlapping matches (noov option). Analyses of the negative controls were done with or without both purge and noov options. The reference set was treated in each case in the same way as the negative controls and further as the recombinant set.

We compared the observed distribution of the E-value obtained with the 1000 negative controls with the expected one for oligonucleotides ranging from 2 to 8 nt. In a first stage we observed a deviation from expectation: more patterns than expected were observed for each specific E-value. Such deviation probably results from an heterogeneity in the composition of the reference set. Consequently, we masked repeated and low complexity sequences using RepeatMasker [59] and resolved this discrepancy between observation and expectation at least for patterns larger than 4 or 5 nucleotides. Figure S1 shows distributions obtained after masking repeats for intervening and extended negative control and for all oligonucleotide sizes and RSAT options. It appeared that the observed distribution fit well to the expected one when noov option is applied and for oligonucleotide size larger than 4 nt for intervening set and 5 nt for extended one. This result is valid whatever the usage of purge option.

We thus applied these valid options (with noov but without purge) to test the recombinant set and retained as possible motifs oligonucleotides larger than 4 and 5 (for intervening and extended recombinant set respectively) showing positive occ-sig.

### Correlation between motif occurrence and crossover rate

This analysis was performed on published genetic map [29]. Physical distance between markers was inferred from version 4.0 of the genome sequence [31] because the most recent version 4.5 is not in agreement with the genetic map order. Crossover rates are calculated by the ratio between genetic length and physical length between consecutive markers located on the same scaffold and separated by at least 45 kb. This threshold was applied for two reasons: i) genetic distances are poorly estimated below 1 cM (corresponding approximately to 45 kb) with the progeny size studied for genetic mapping, ii) accurate estimation of pattern occurrence also relies on sufficient DNA fragments. The occurrences of the tested oligonucleotides were standardized to 10 kb fragments to allow comparisons between fragments of various size (between 45 and 425 kb). Pearson correlation coefficient were calculated and tested after Benjamini and Hochberg correction for multiple testing [60] using R 2.13.1 [61].

## Supporting Information

Figure S1 **Analysis of the fit to the theoretical model.** Set of non recombinant fragments was used to generate 1000 negative controls comprising the same number of sequence than the recombinant set and sequences of similar size. These sequences were tested as is or extended by 40 nt on each side to generate similar set than intervening or extended set respectively. These negative controls as well as the reference set were masked for repeats and low complexity sequences. The negative controls were then tested against the reference set to search for significant patterns of size 2 to 8 nt with various RSAT option (with or without noov and purge options). The number of significant patterns is counted for each set and the frequency is plotted for each significance value. The red curve shows the expected number of false positives per dataset. False positives observed follow this curve when noov option is applied and when oligonucleotides are 4 or more nucleotides long for intervening set and 5 nt or more for extended set.
(PDF)
