# Supplementary material for: Fine Scale Analysis of Crossover and Non-Crossover and Detection of Recombination Sequence Motifs in the Honeybee (Apis mellifera)
Source: PLoS One. 2012 May 2;7(5):e36229. doi: 10.1371/journal.pone.0036229 (PMC3342173; doi:10.1371/journal.pone.0036229)
Supplement: Figure S1 — Analysis of the fit to the theoretical model. Set of non recombinant fragments was used to generate 1000 negative controls comprising the same number of sequence than the recombinant set and sequences of similar size. These sequences were tested as is or extended by 40 nt on each side to generate similar set than intervening or extended set respectively. These negative controls as well as the reference set were masked for repeats and low complexity sequences. The negative controls were then tested against the reference set to search for significant patterns of size 2 to 8 nt with various RSAT option (with or without noov and purge options). The number of significant patterns is counted for each set and the frequency is plotted for each significance value. The red curve shows the expected number of false positives per dataset. False positives observed follow this curve when noov option is applied and when oligonucleotides are 4 or more nucleotides long for intervening set and 5 nt or more for extended set. (PDF) [file pone.0036229.s001.pdf]

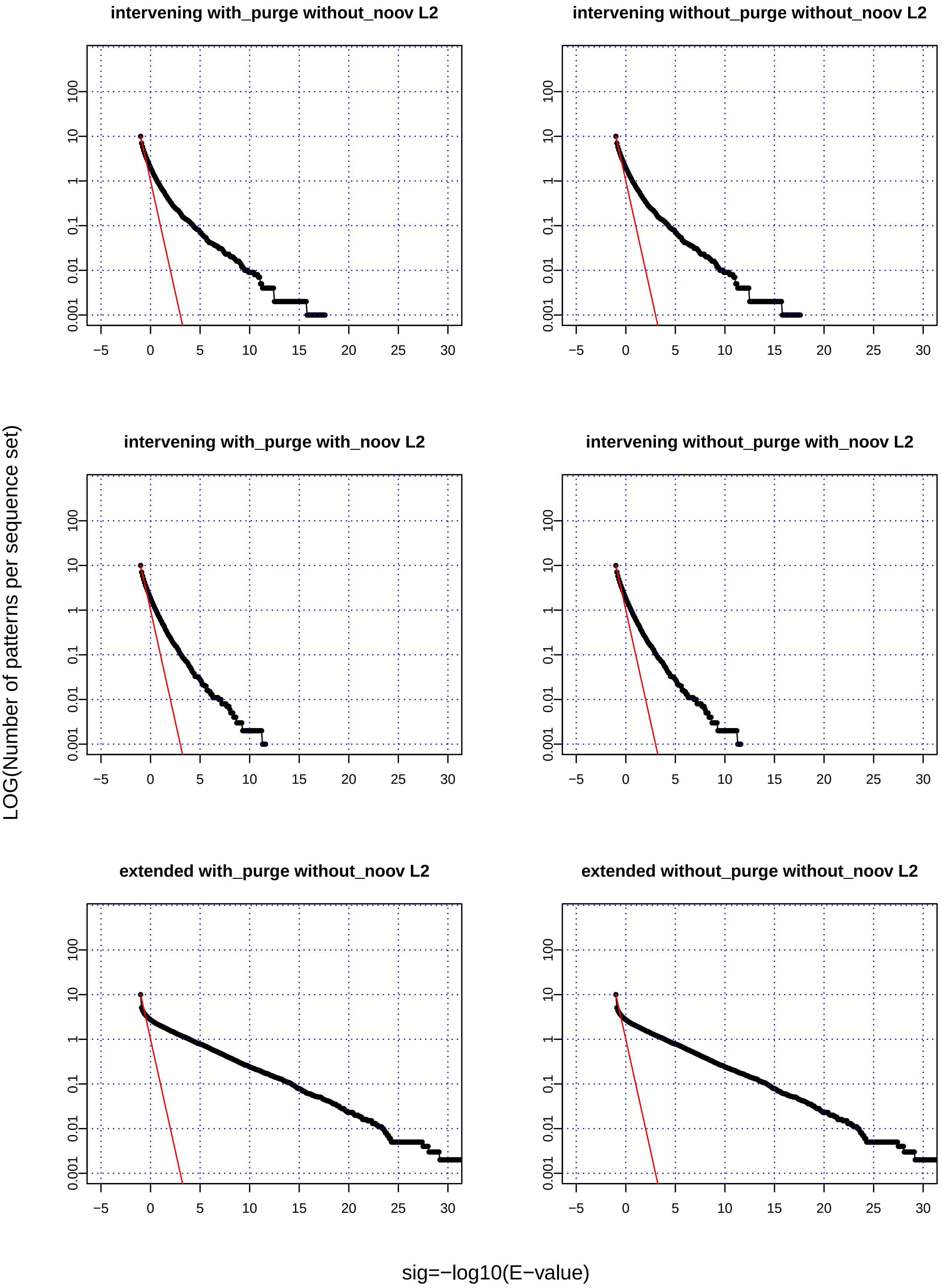

intervening with_purge without_noov L2
intervening without_purge without_noov L2
intervening with_purge with_noov L2
intervening without_purge with_noov L2
extended with_purge without_noov L2
extended without_purge without_noov L2
LOG(Number of patterns per sequence set)
sig=−log10(E−value)
0.001
0.01
0.1
1
10
100
−5
0
5
10
15
20
25
30

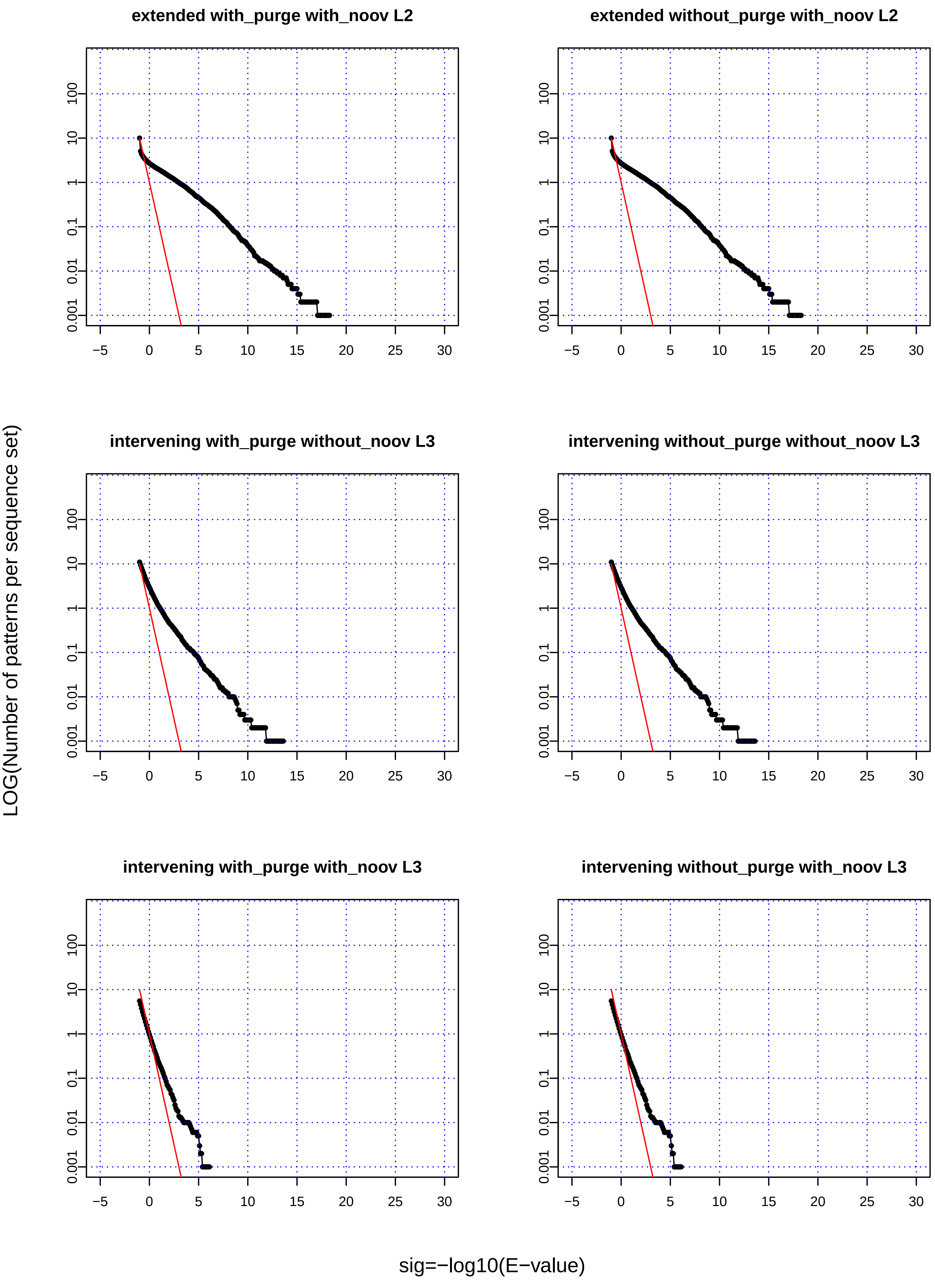
extended with_purge with_noov L2
extended without_purge with_noov L2
intervening with_purge without_noov L3
intervening without_purge without_noov L3
intervening with_purge with_noov L3
intervening without_purge with_noov L3
100
10
1
0.1
0.01
0.001
−5
0
5
10
15
20
25
30
LOG(Number of patterns per sequence set)
sig=−log10(E−value)

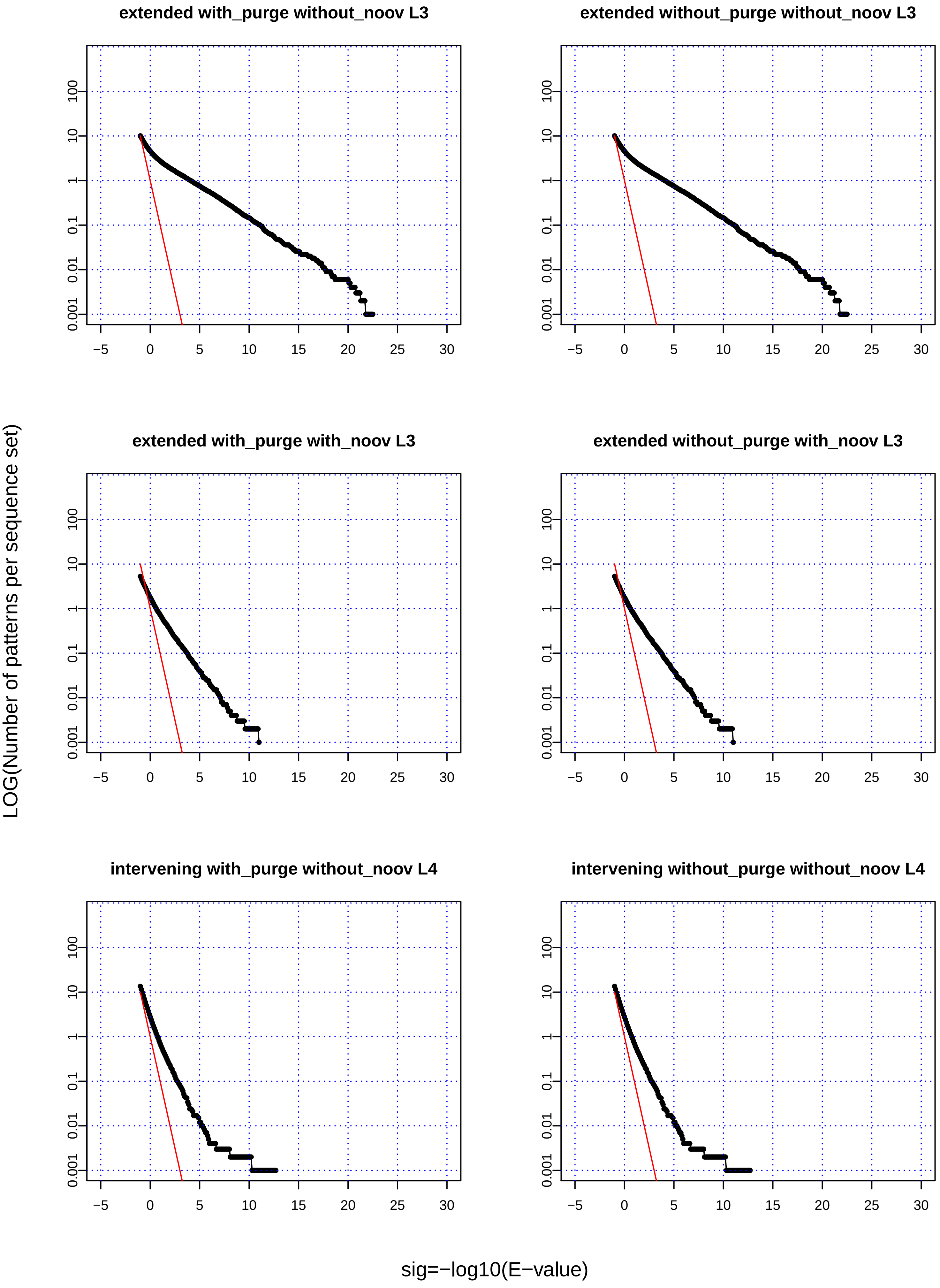

extended with_purge without_noov L3
extended without_purge without_noov L3
extended with_purge with_noov L3
extended without_purge with_noov L3
intervening with_purge without_noov L4
intervening without_purge without_noov L4
0.001 0.01 0.1 1 10 100
−5 0 5 10 15 20 25 30
LOG(Number of patterns per sequence set)
sig=−log10(E−value)

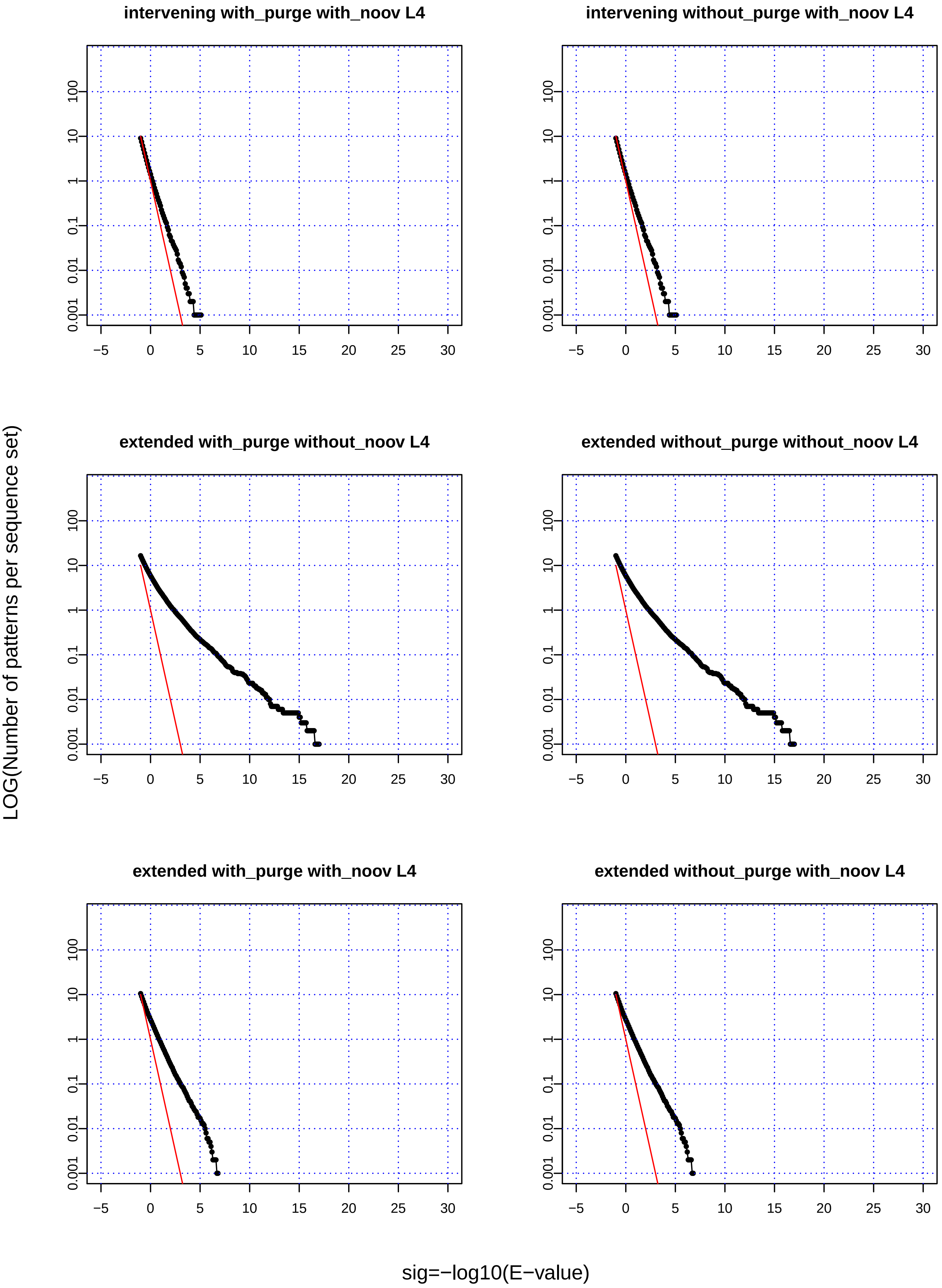
intervening with_purge with_noov L4
intervening without_purge with_noov L4
extended with_purge without_noov L4
extended without_purge without_noov L4
extended with_purge with_noov L4
extended without_purge with_noov L4
LOG(Number of patterns per sequence set)
sig=−log10(E−value)
0.001
0.01
0.1
1
10
100
−5
0
5
10
15
20
25
30

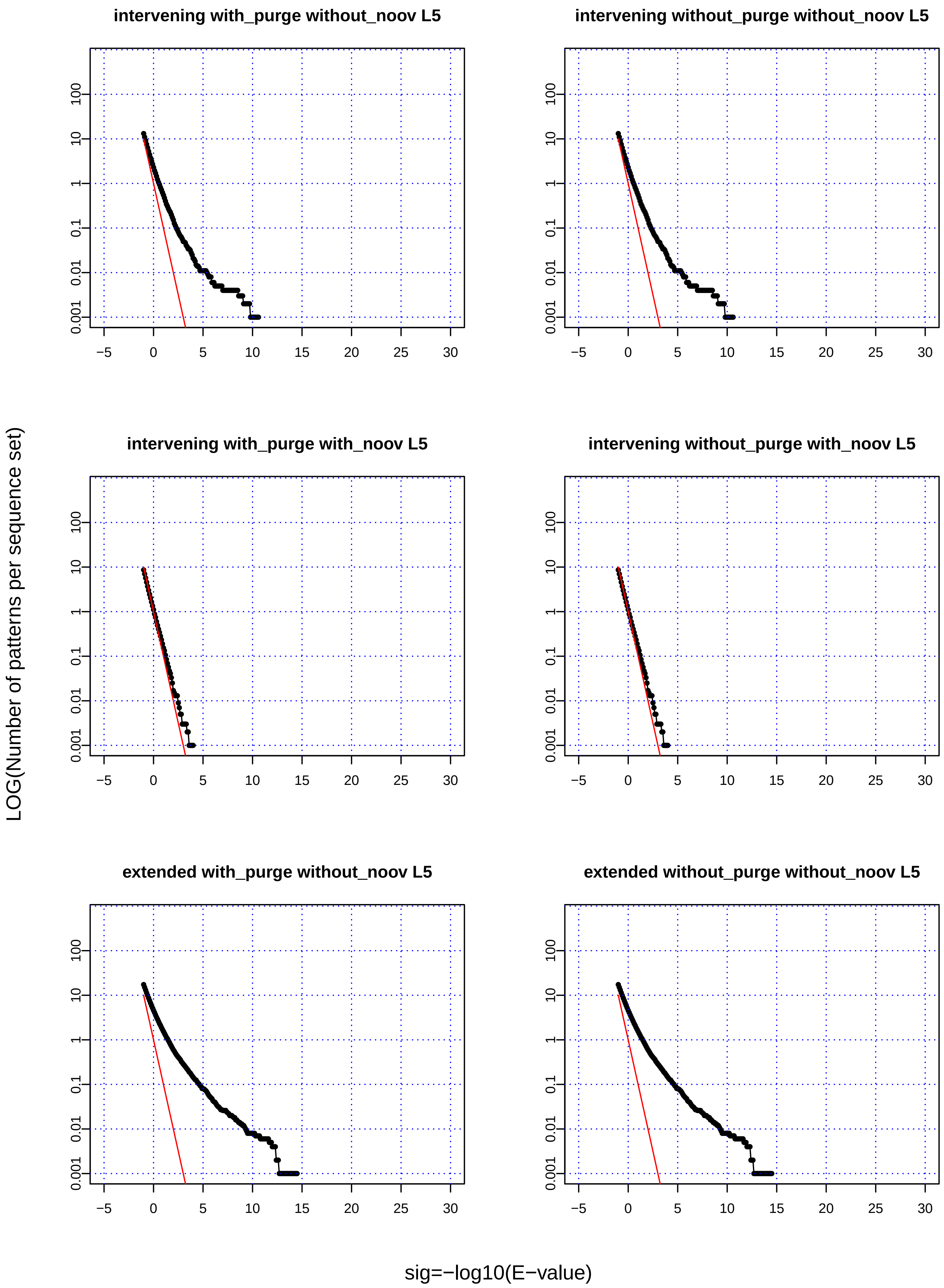

intervening with_purge without_noov L5
intervening without_purge without_noov L5
intervening with_purge with_noov L5
intervening without_purge with_noov L5
extended with_purge without_noov L5
extended without_purge without_noov L5
0.001
0.01
0.1
1
10
100
−5
0
5
10
15
20
25
30
LOG(Number of patterns per sequence set)
sig=−log10(E−value)

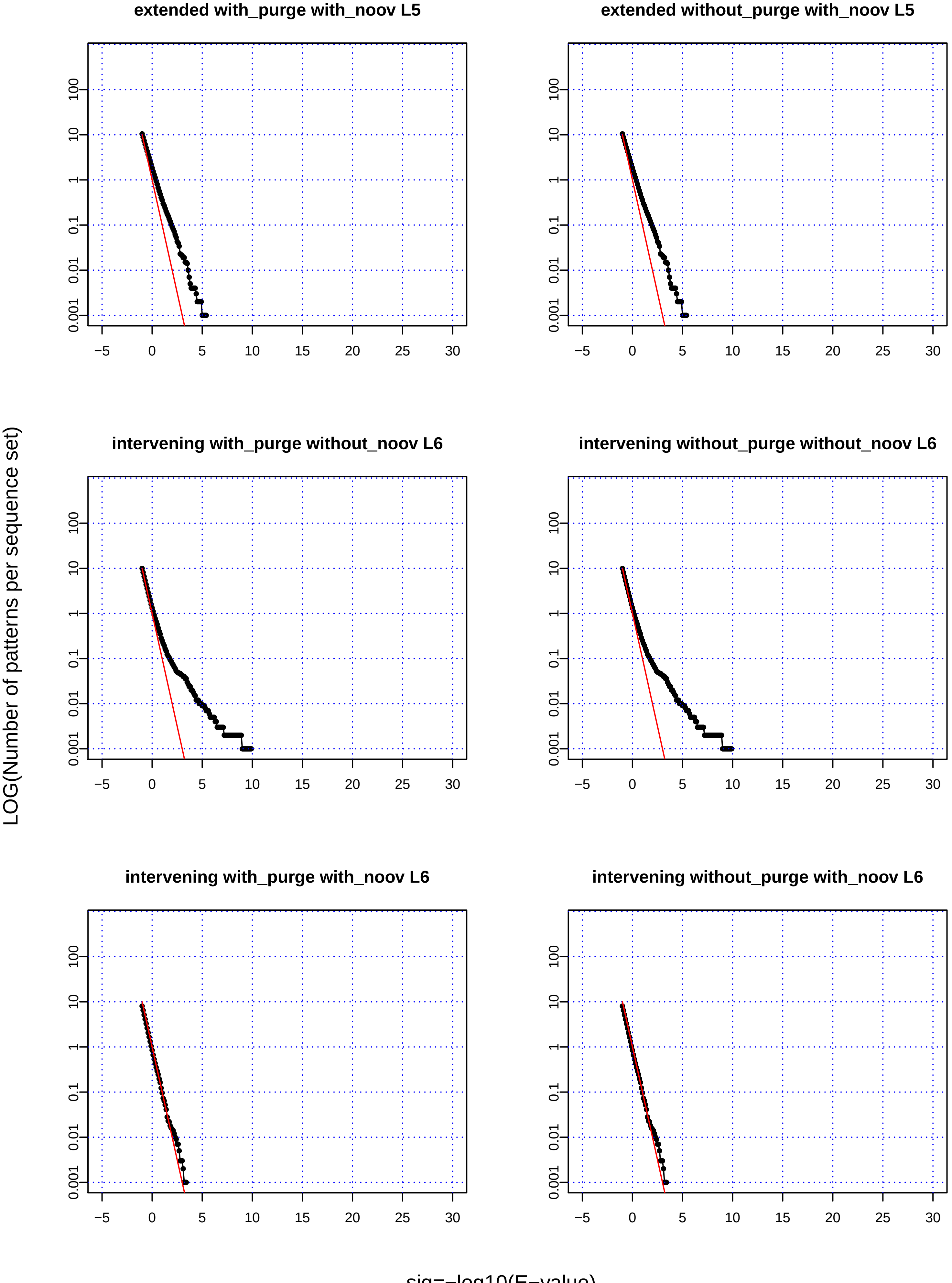

extended with_purge with_noov L5
extended without_purge with_noov L5
intervening with_purge without_noov L6
intervening without_purge without_noov L6
intervening with_purge with_noov L6
intervening without_purge with_noov L6
0.001
0.01
0.1
1
10
100
−5
0
5
10
15
20
25
30
LOG(Number of patterns per sequence set)
sig=−log10(E−value)

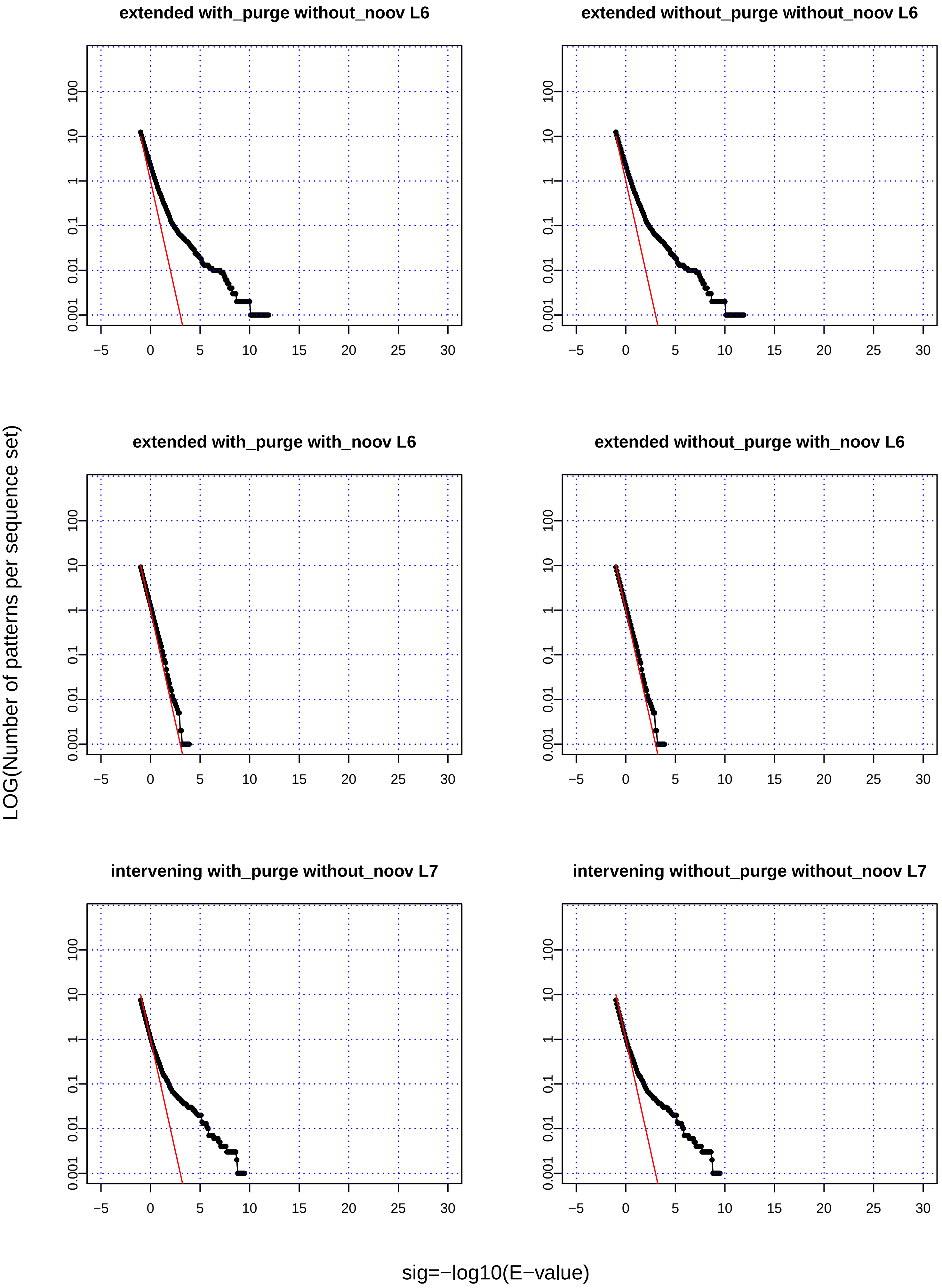
extended with_purge without_noov L6
extended without_purge without_noov L6
extended with_purge with_noov L6
extended without_purge with_noov L6
intervening with_purge without_noov L7
intervening without_purge without_noov L7
LOG(Number of patterns per sequence set)
sig=−log10(E−value)
0.001
0.01
0.1
1
10
100
−5
0
5
10
15
20
25
30

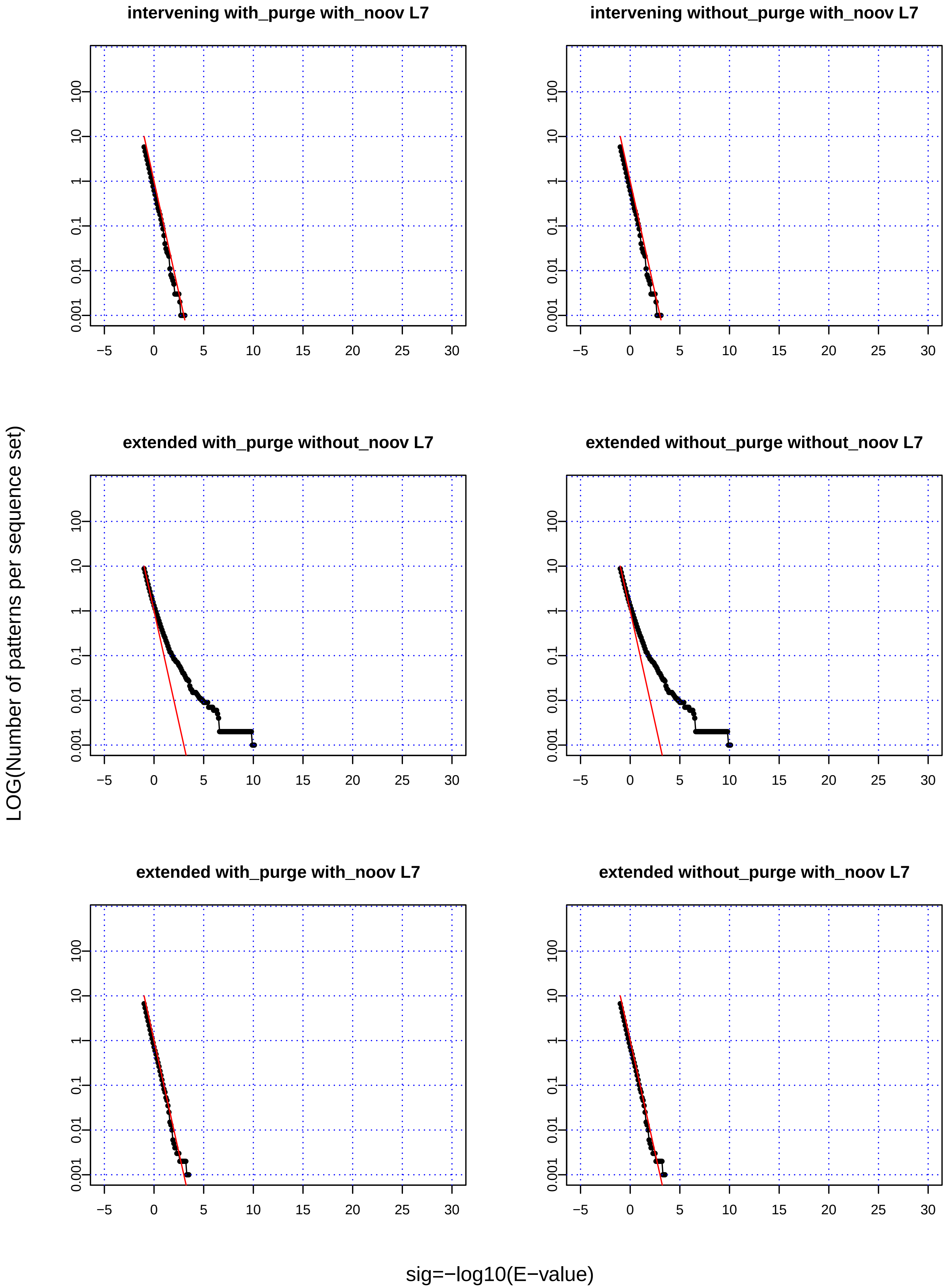

intervening with_purge with_noov L7
intervening without_purge with_noov L7
extended with_purge without_noov L7
extended without_purge without_noov L7
extended with_purge with_noov L7
extended without_purge with_noov L7
LOG(Number of patterns per sequence set)
sig=−log10(E−value)

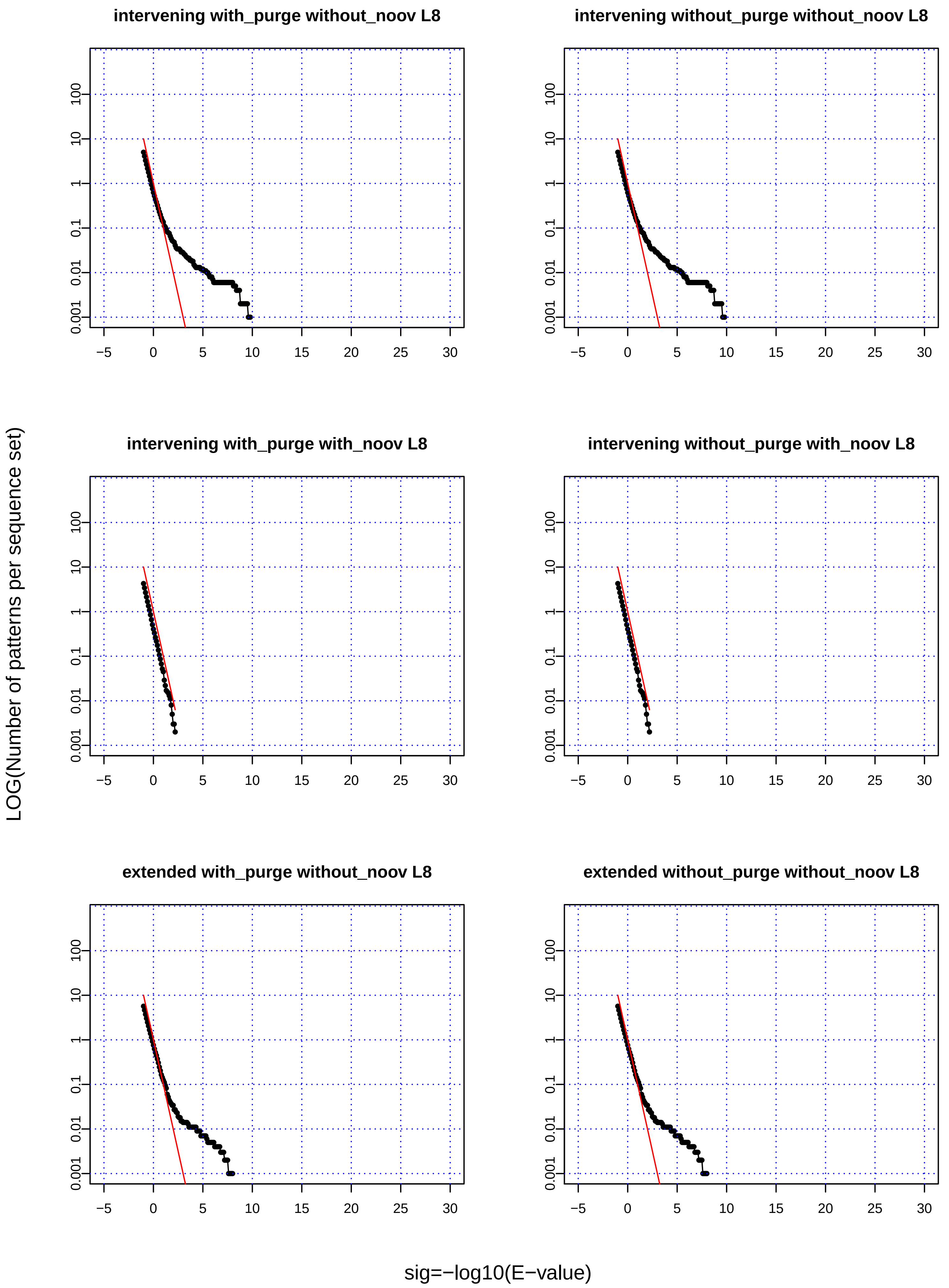
intervening with_purge without_noov L8
intervening without_purge without_noov L8
intervening with_purge with_noov L8
intervening without_purge with_noov L8
extended with_purge without_noov L8
extended without_purge without_noov L8
0.001
0.01
0.1
1
10
100
−5
0
5
10
15
20
25
30
LOG(Number of patterns per sequence set)
sig=−log10(E−value)

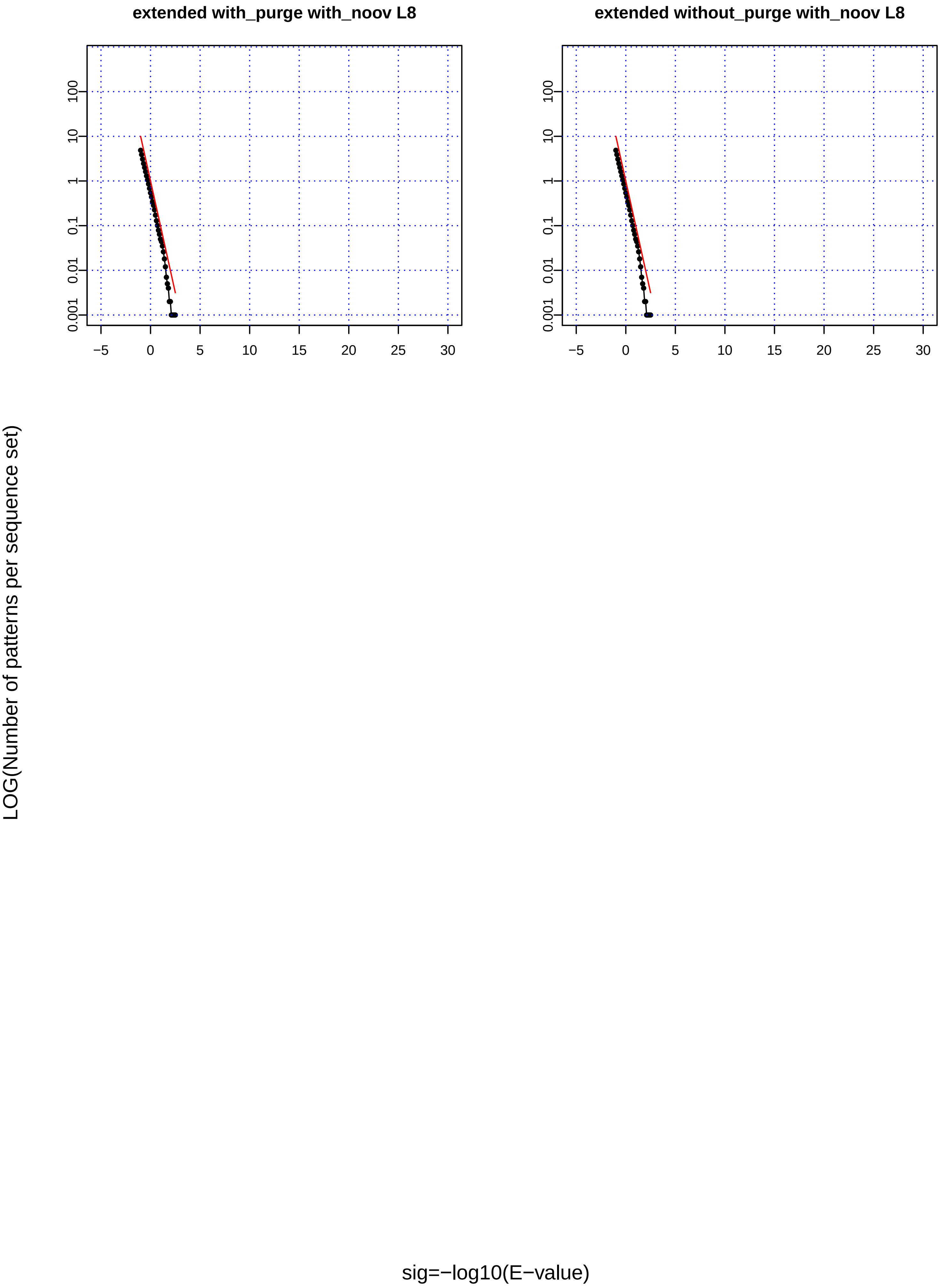
extended with_purge with_noov L8
extended without_purge with_noov L8
0.001
0.01
0.1
1
10
100
−5
0
5
10
15
20
25
30
LOG(Number of patterns per sequence set)
sig=−log10(E−value)
